# Endometrial cancer (EC) derived G3BP1 overexpression and mutant promote EC tumorigenesis and metastasis via SPOP/ERα axis

**DOI:** 10.1186/s12964-023-01342-7

**Published:** 2023-10-30

**Authors:** Yidong Ge, Jiabei Jin, Gun Chen, Jinyun Li, Meng Ye, Xiaofeng Jin

**Affiliations:** 1 Department of Radiotherapy and Chemotherapy, The First Hospital of Ningbo University, Ningbo University, Ningbo, 315010 China; 2 The Affiliated People’s Hospital of Ningbo University, Ningbo, 315040 China; 3 Department of Biochemistry and Molecular Biology, Zhejiang Key Laboratory of Pathophysiology, Health Science Center, Medical School of Ningbo University, Ningbo University, Ningbo, 315211 Zhejiang China

**Keywords:** Ras-GTPase-activating protein binding protein 1, Speckle-type POZ protein, Estrogen receptor alpha, Endometrial cancer, Fulvestrant

## Abstract

**Background:**

Ras-GTPase-activating protein binding protein 1 (G3BP1) is an oncogenic factor, which highly expressed in a variety of cancers. In recent years, G3BP1 has been reported to promote the development of prostate cancer by inhibiting the degradation of AR through inhibiting SPOP. However, whether G3BP1 contributes in a similar manner to the abnormal accumulation of ERα, which is also an important target for hormone therapy, remains unknown. This article addresses this issue and explores potential mechanisms.

**Methods:**

Bioinformatics tools were used for G3BP1 expression analysis, survival analysis, and clinical association analysis. Immunohistochemical staining was used to examine the correlation between G3BP1 and ERα in EC patients. In addition, western blot and co-immunoprecipitation were used to detect the half-life of G3BP1 and mutant, and the effect of G3BP1 and mutant on the ubiquitination and degradation of ERα mediated by SPOP. Then, the oncogenic functions of G3BP1 dependent on the SPOP/ERα axis were determined by CCK8 cell proliferation assay, colony formation assay and cell migration assay. Finally, we established the EC cells treated or untreated with fulvestrant, exploring the possibility of fulvestrant combined with the reduction of G3BP1 to improve the efficacy of fulvestrant.

**Results:**

G3BP1 is abnormally high expressed and characterized by high-frequency mutation in EC. In addition, there is a positive correlation between G3BP1 protein and ERα protein. Mechanistically, both G3BP1 and mutant, the latter is displaying the longer half-life, competitively bind SPOP with ERα, thereby inhibiting SPOP-mediated ubiquitination and degradation of ERα. Functionally, G3BP1 and mutant promote the proliferation and migration of EC cells by regulating the G3BP1/SPOP/ERα axis. However, fulvestrant can reverse the cancer-promoting effects of G3BP1 and mutant.

**Conclusions:**

G3BP1 and its mutant positively regulate ERα signaling pathway by inhibiting SPOP-mediated ubiquitination and degradation of ERα, indicating the promising effect of fulvestrant on the suppression the occurrence and development of EC with high expressed G3BP1 and G3BP1 mutants.

Video Abstract

**Supplementary Information:**

The online version contains supplementary material available at 10.1186/s12964-023-01342-7.

## Background

Endometrial cancer (EC) is one of the most common gynecological malignancies and the sixth most common malignant disease in the world [1]. At present, EC patients with progesterone receptor and/or estrogen receptor positive could be considered for endocrine therapy [2]. Progesterone is commonly used in the treatment of well-differentiated relapsed EC patients or patients with advanced EC requiring fertility preservation [2]. It prolong progression-free survival (PFS) but has no influence on overall survival (OS) [2]. Tamoxifen, a selective estrogen receptor modulator, competes with estrogen for estrogen receptor alpha (ERα), thereby inhibiting the development of EC [3]. However, some studies have shown that tamoxifen can enhance ERα transcriptional activity through MAPK signaling pathway leading to EC proliferation and carcinogenesis [3]. Therefore, endocrine therapy efficacy of EC needs to be further improved.

ERα is an important nuclear transcription factor that mediates estrogen-responsive cancers, including EC [4]. Upon binding to estrogen, ERα forms dimers and translocated into the nucleus, where it summons co-activators or co-repressors to the estrogen response element (ERE) on the target gene promoter, thereby activating or inhibiting transcription [5]. Studies have shown that ERα protein is highly expressed in EC, and anti-ERα therapy has been shown to be effective in EC [2]. However, the mechanism of the abnormally high expression of ERα in EC remains unclear.

Ras-GTPase-activating protein binding protein 1 (G3BP1) is a protein of 466 amino acid whose primary biological function is to promote the assembly of stress granules in the cytoplasm of eukaryotic cells in response to certain environmental stresses [6]. It has been reported that G3BP1 could competitively inhibit the binding of Speckle-type POZ protein (SPOP) to androgen receptor (AR) [7]. As a substrate adapter protein of Cullin3-RING ubiquitin ligase (CRL3), SPOP recruits substrates to CRL3 for ubiquitination and proteasome degradation [8]. The substrates of SPOP include AR, ERα, BET protein and some other important oncoproteins [9]. In EC, SPOP specifically recognizes ERα and promotes its degradation through the ubiquitin–proteasome system (UPS), thus inhibiting the transcriptional activity of ERα, which inhibits the proliferation, metastasis and invasion of EC [10].

Herein, we demonstrated that G3BP1 protein is highly expressed and is positively correlated with ERα in EC tissues. Mechanistically, we found that G3BP1 and its EC-associated mutant dramatically inhibit SPOP-mediated ubiquitination and degradation of ERα. Functionally, we found that G3BP1 and its mutant promote EC cell proliferation and metastasis, and this effect could be reversed by the fulvestrant, an ERα antagonist commonly used in clinical, indicating a promising idea for improving the efficacy of EC treatment.

## Materials and methods

### Cell culture

HEK 293T, HEC-1-A and AN3-CA cells were purchased from the American Type Culture Collection. HEK 293T and HEC-1-A cells were cultured in DMEM supplemented with 10% FBS and 1% penicillin–streptomycin solution. AN3-CA cells were cultured in MEM supplemented with 10% FBS and 1% penicillin–streptomycin solution. All cells were grown at 37 °C with 5% CO2.

### siRNA and plasmids transfection

Cells were transfected with siRNA (Tsingke Biotechnology, Hangzhou, China) using Lipo6000™ or Lipo8000™ Transfection Reagent (Beyotime, shanghai, China) as described by the manufacturer's instructions. The siRNA oligonucleotide sequences of G3BP1 and SPOP are shown in Table S1.

### Antibodies and reagents

The following antibodies and reagents were used: G3BP1 (Proteintech, #13,057–2-AP, Wuhan, China), ERα (Proteintech, #21,244–1-AP, Wuhan, China), SPOP (Proteintech, #16,750–1-AP, Wuhan, China), GAPDH (Abclonal, #AC001, Wuhan, China), FLAG (MBL, #M185-7, Tokyo, Japan), Myc (MBL, #M192-7, Tokyo, Japan), HA (MBL, #M180-7, Tokyo, Japan), Anti-Rabbit (Abclonal, #AS014, Wuhan, China), Goat Anti-Rabbit (Proteintech, # SA00013-4, Wuhan, China), anti-Flag M2 agarose beads (Smart-Lifesciences, #SA042100, Changzhou, CHINA), MG-132 (Selleckchem, #S2619, Houston, USA), CHX (MCE, #HY-B0713, Shanghai, China).

### Bioinformatics analysis

The mRNA expression (FPKM) and clinicopathological features were derived from The Cancer Genome Atlas (TCGA, https://portal.gdc.cancer.gov/) and the GSE17025 of GEO cohort (https://www.ncbi.nlm.nih.gov/gds/). The protein expression and clinicopathological features were derived from Clinical Proteomic Tumor Analysis Consortium (CPTAC, https://cptac-data-portal.georgetown.edu/). The FPKM was transformed to TPM [Log2 (FPKM + 1)]. And the analysis was conducted by the “BiocManager”, “heatmap”, “limma”, “ggplot2” “survival” and “survminer” packages in R software (version 4.2.1). Pearson correlation analysis was used to screen out the genes significantly related to G3BP1 expression. Gene Ontology (GO) and Kyoto Encyclopedia of Genes and Genomes (KEGG) analyzed the gene sets and their biological functions based on CPTAC. The mutation frequency and mutation site of G3BP1 were derived from TCGA and cBioPortal cohort (http://www.cbioportal.org/).

### Co-immunoprecipitation

HEK 293T cells were transfected the indicated constructs. After transfection for 36 h, cells were treated with 30 μM MG132 for 8 h and then lysed in Medium RIPA lysis buffer (Solarbio, Beijing, China). Next, anti-Flag M2 agarose beads (Sigma) were added to the cell lysates overnight. Subsequently, the bound beads are then washed 3 times with BC100 buffer. The bound proteins were eluted using FLAG-peptide (Sigma)/BC100, and added with SDS-PAGE solution to boil for Western blot.

### GST pull-down assays

The GST fusion protein was fixed on glutathione-Sepharose beads (Amersham Biosciences). After washing with a pull-down buffer, the beads were incubated with recombinant FLAG-labeled proteins for 2 h. The beads are then washed 5 times with a binding buffer and re-suspended in the sample buffer. The binding proteins were added with SDS-PAGE solution to boil for Western blot.

### Western blot

Cell lysates or immunoprecipitations were subjected to SDS-PAGE and protein transfer to nitrocellulose membranes (GE Healthcare, Little Chalfont, UK). The membrane was blocked in Tris-buffered saline (pH 7.4) with 5% skim milk and 0.1% Tween-20, incubated overnight with primary antibody at 4°, then incubated with secondary antibody for 1 h. Proteins of interest were visualized using the ECL Chemiluminescence System (Santa Cruz, Santa Cruz, CA, USA). WB was performed for 2–3 times from at least two independent experiments and representative pictures were shown.

### Real-time quantitative polymerase chain reaction (qRT-PCR)

Total RNA was isolated from cells using Trizol reagent (Tiangen, China). cDNA synthesis was performed using the HiScript® II 1st Strand cDNA Synthesis Kit (Vazyme, China) according to manufacturer's instructions. PCR amplification was performed using SYBR Green PCR Master Mix Kit (Vazyme, China). All quantization were normalized to the level of endogenous control GAPDH. The primer sequences for qRT-PCR are shown in Table S1.

### Immunohistochemistry

One hundred twenty pairs of paraffin-embedded EC tissues were cut to 4 μm thickness and roasted at 65℃ for 3 h. Then, roasted tissue sections were dewaxed with xylene and rehydrated with ethanol. Antigen repair is performed by heating tissue sections in a pressure cooker in Ethylene Diamine Tetra acetic Acid (EDTA) antigen repair buffer, PH9.0 (Beyotime, Shanghai, P0085) for 10 min and then cooling them naturally to room temperature. Then, the endogenous peroxidase was inactivated by treatment with 3% hydrogen peroxide for 20 min, permeated with 1% Triton X-100 (Solarbio, T8200, Beijing, China) for 30 min, blocked with 10% donkey serum for 15 min, and incubated at 4℃ overnight with G3BP1 antibody (Proteintech, #13,057–2-AP, Wuhan, China) (1:400) or ERα antibody (Proteintech, #21,244–1-AP, Wuhan, China) (1:200). The next day, sections were incubated with secondary antibody (SA00001-2, Proteintech, Wuhan, China) (1:500) at room temperature for 1 h, stained with DAB assay kit (Solarbio, G1212, Beijing, China) for 5–7 min, and stained with hematoxylin (Solarbio, G4070, Beijing, China) for 10s.

All samples were reviewed by two independent pathologists experienced in IHC evaluation who had no knowledge of clinical outcomes in these patients. They assessed the percentage of positively stained cells and staining intensity to semi-quantitatively determine G3BP1 and ERα expression. The percentage score of positive staining cells was as follows: 0, < 10%; 1, 10%-50%; 2, > 50%. The staining intensity is graded as follows: 0 (no or weak staining = light yellow), 1 (moderate staining = yellow brown), and 2 (strong staining = brown). The total score of G3BP1 and ERα expression was the sum of the percentage of cells and the staining intensity score for positive staining, with the total score ranging from 0 to 4. For statistical analysis, the final score was a combination of independent scores assigned by the two pathologists reported in this study. Any differences in scores were resolved through discussion between the two pathologists.

### CCK8 cell proliferation assay

The cell proliferation rate was determined using Cell Counting Kit-8 (CCK-8) (Dojindo Laboratories, Japan) according to the manufacturer's instructions. AN3-CA and HEC-1-A cells were inoculated into the 96-well plate with a density of 1500 cells per well (three wells in each group). During 0–5 d culture, 10 μl CCK-8 solution was added at the same time every day and cultured for 2 h. After these, OD values of each well were measured at 450 nm using a microplate absorbance instrument (Bio-Rad, US). Each assay was repeated three times.

### Colony formation assay

AN3-CA and HEC-1-A cells were inoculated in 6-well plates with 2000 cells per well (three wells in each group). After 10–15 days of culture, the cells were fixed in 4% polymethanol at 4° for 30 min and then stained with crystal violet solution (G4070, Solarbio, China) for 15–20 min. The images of cell colonies were captured by smartphone and analyzed by Image J software.

### Cell migration assay

Transwell chambers (8.0 μm, 3342, Corning, USA) were also used to measure cell migration. 40,000 cells were seeded in each upper chamber with 200 μl fresh medium without FBS, and 500 μl medium with 10% FBS in each lower chamber. Three duplicate wells were set up for each group. After 24 h, the cells were fixed in 4% polymethanol at 4° for 30 min and then stained with crystal violet solution (G4070, Solarbio, China) for 15–20 min. Clean the upper chamber and count the number of cells on the lower surface of the membrane.

### Plasmid constructions

The expression vectors for SPOP were described previously [11]. The G3BP1 cDNA was purchased from Miaolingbio (Wuhan, China) and was subcloned into pCMV-FLAG and pCMV-HA expression vectors. The FLAG-ERα was purchased from Miaolingbio (Wuhan, China) and was subcloned into pCMV-Myc and pCMV-HA expression vectors. The G3BP1 Q392* constructions is subcloned from FLAG-G3BP1 and was subcloned into pCMV-Myc and pCMV-FLAG expression vectors. All constructs were validated by DNA sequencing.

### Statistical analysis

Statistical analysis data were analyzed with student's t-test and presented as mean value ± standard deviation of triplicate measurements in three separate experiments. Statistical calculations were performed using GraphPad Prism 8 software. The images were analyzed and quantified using ImageJ software. * indicates *P* < 0.05; ** indicates *P* < 0.01; *** indicates *P* < 0.001; **** indicates *P* < 0.0001.

## Results

### G3BP1 protein is highly expressed and frequently mutated in endometrial carcinoma

**Fig. 1:**
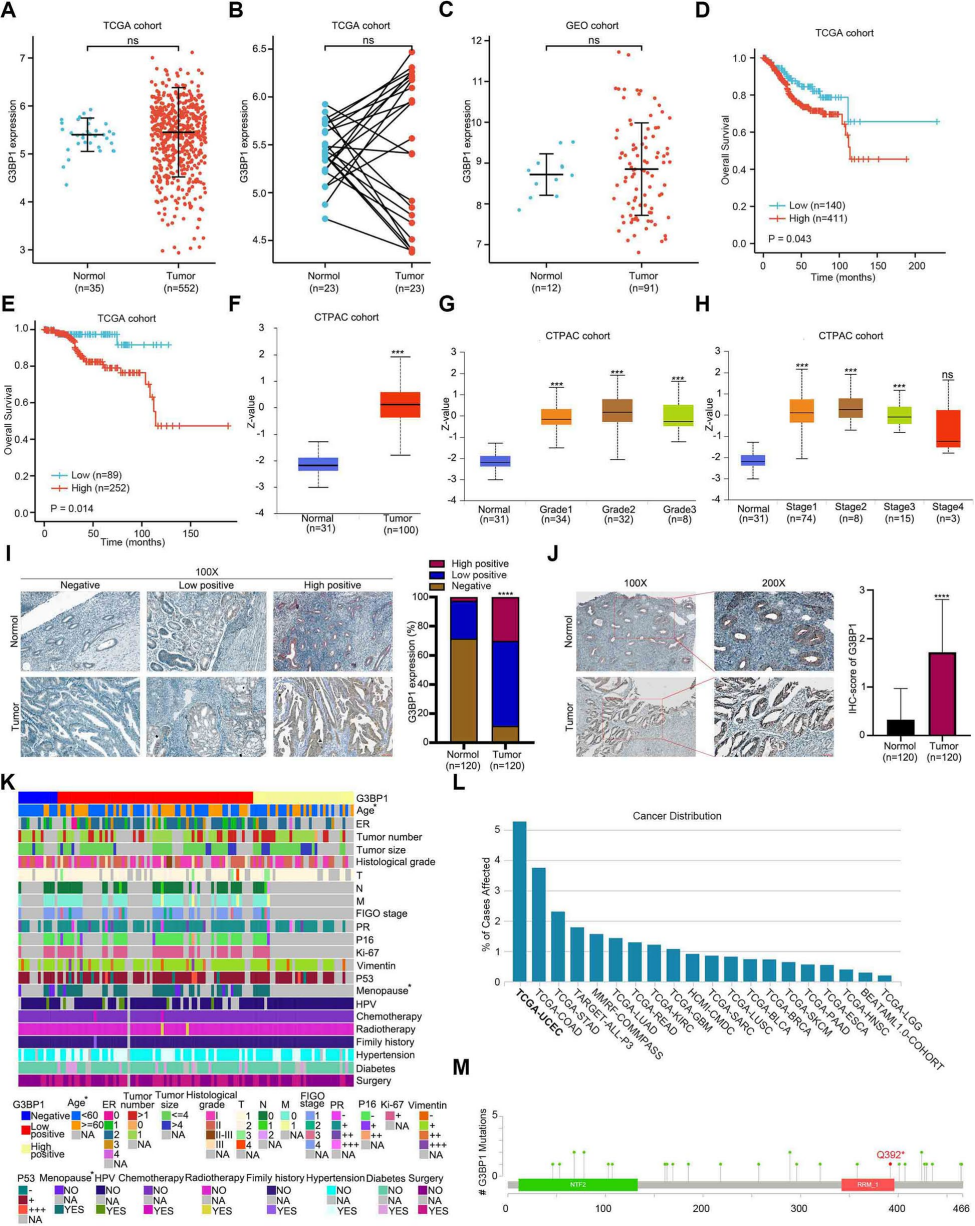
G3BP1 protein is highly expressed and frequently mutated in endometrial carcinoma. **A**, **B** Relative expression of G3BP1 mRNA in normal endometrial tissues and EC tissues from the TCGA cohort (Normol, *n* = 35; Tumor, *n* = 552). **C** Relative expression of G3BP1 mRNA in normal endometrial tissues and EC tissues from the GEO cohort (GSE17025) (Normol, *n* = 12; Tumor, *n* = 91). **D** Kaplan–Meier survival curves of EC with high and low G3BP1 mRNA expression from the TCGA cohort (Low, *n* = 140; High, *n* = 411). **E** Kaplan–Meier survival curves of stage 1 EC samples with high and low G3BP1 mRNA expression from the TCGA cohort (Low, *n* = 89; High, *n* = 252). **F** Relative expression of G3BP1 protein in normal endometrial tissues and EC tissues from the CPTAC cohort (Normol, *n* = 31; Tumor, *n* = 100). **G** Relative expression of G3BP1 protein in normal endometrial tissues and different grades EC tissues from the CPTAC cohort (Normol, *n* = 31; Grade 1, *n* = 34; Grade 2, *n* = 32; Grade 3, *n* = 8). **H** Relative expression of G3BP1 protein in normal endometrial tissues and different stages EC tissues from the CPTAC cohort (Normol, *n* = 31; Stage 1, *n* = 74; Stage 2, *n* = 8; Stage 3, *n* = 15; Stage 4, *n* = 3). **I** Immunohistochemical staining showed representative images of different levels of G3BP1 expression in EC tissues and normal tissues, and the proportion of three different staining intensities. (Normal, *n* = 120; Tumor, *n* = 120), Scale bar, 10 μm. **J** Immunohistochemical staining showed representative images of G3BP1 expression levels in EC and normal endometrial tissue, and immunohistochemical scores were calculated. (Normal, *n* = 120; Tumor, *n* = 120), Scale bar, 10 μm. **K** Heat map of clinical characteristics of 120 patients with EC. (**L**) The TCGA cohort shows the distribution of G3BP1 mutations in different cancers. **M** Schematic diagram of the G3BP1 protein shows the location of mutations found in the EC TCGA cohort. Data are shown as mean ± SD (*n* = 3). *P < 0.05, **P < 0.01, ***P < 0.001, ****P < 0.0001

Recently, G3BP1 protein has been gradually recognized as an oncoprotein, which plays an important role in the occurrence and development of various tumors. However, whether G3BP1 is still a carcinogenic protein in EC remains poorly understood. To address this issue, we used bioinformatics to analyze the role of G3BP1 in EC samples from the TCGA, GEO, and CPTAC cohorts. Through unpaired and paired analysis of 587 samples from TCGA, we found that there is no difference in the expression of G3BP1 in EC and normal tissues at the mRNA level (Fig. 1A, B). Similarly, samples from GEO cohort also suggest that G3BP1 mRNA is not significantly highly expressed in EC tissues (Fig. 1C). However, the expression of G3BP1 mRNA is associated with OS in patients with EC. The higher level of G3BP1 mRNA, the lower OS of patients (Fig. 1D, S1A). In addition, the difference was particularly pronounced in stage I EC (Fig. 1E). At the protein level, G3BP1 protein was significantly higher in EC than that in normal tissue, as indicated by the CPTAC cohort (Fig. 1F). Through further investigation of CPTAC by UALCAN (http://ualcan.path.uab.edu/index.html), we found that the level of G3BP1 protein is correlated with the clinical stage and grade of patients with EC (Fig. 1G, H). The higher expression of G3BP1 protein in EC is associated with higher clinical stage and grade. Moreover, immunohistochemical staining was performed on 120 pairs of paraffin-embedded EC tissues to confirm the expression of G3BP1 protein. Compared with normal endometrial tissues, G3BP1 protein was significantly upregulated in EC tissues, presenting a large number of highly positive staining (Fig. 1I, J). Moreover, through the analysis of clinical information from these 120 pairs of EC specimens, we found that the expression of G3BP1 protein was correlated with age and menopausal status of patients (Fig. 1K, Table 1). In addition, the TCGA cohort showed that G3BP1 transcriptional expression level was concerned with the grade of EC (Fig. S1B), while the CPTAC cohort showed that G3BP1 protein was not related to the clinical features recorded (Fig. S1C). Finally, according to the statistics of TCGA cohort, among all cancers, the mutation frequency of *G3BP1* in EC is the highest, coming to 5.27% (27/512), including missense mutation, synonymous mutation, and nonsense mutation (Fig. 1L). It is noted that there are only two kinds of nonsense mutants in EC, namely G3BP1 Q68* and G3BP1 Q392* (Fig. 1M). But prognostic analysis of EC in the TCGA cohort showed no difference in OS between the G3BP1 mutant and the wild-type G3BP1 (Fig. S1A). Since the interaction domain of G3BP1 with SPOP is incomplete in G3BP1 Q68* [7], we pay special attention to G3BP1 Q392*.

**Table 1:** Correlation between the clinicopathological characteristics and G3BP1 expression

**Characteristic**	**Number**	**G3BP1, n (%)**				
		**Negative**	**Low positive**	**High positive**	**χ2**	***P* value**
**Age (years), n (%)**					6.977	**0.0305**
< = 60	69	13(11.82%)	36(32.73%)	20(18.18%)		
> 60	41	1(0.91%)	29(26.36%)	11(10%)		
**Height (cm), n (%)**					2.740	0.254
< = 160	71	7(6.67%)	45(42.86%)	19(18.10%)		
> 160	34	7(6.67%)	17(16.19%)	10(9.52%)		
**Weight (kg), n (%)**					3.724	0.1553
< = 60	46	6(5.71%)	23(21.90%)	17(16.19%)		
> 60	59	8(7.62%)	39(37.14%)	12(11.43%)		
**BMI (kg/m**^**2**^**), n (%)**					0.8824	0.6433
< = 24	48	7(6.67%)	26(24.76%)	15(14.29%)		
> 24	57	7(6.67%)	36(34.29%)	14(13.33%)		
**Tumor number**					0.1297	0.9372
Single	51	6(7.32%)	30(36.59%)	15(18.29%)		
Multiple	31	3(3.66%)	18(21.95%)	10(12.20%)		
**Tumor size (cm), n (%)**					2.903	0.2342
< = 4	61	8(10.26%)	37(47.44%)	16(20.51%)		
> 4	17	1(1.28%)	8(10.26%)	8(10.26%)		
**Histological grade, n (%)**					3.284	0.5114
G1	43	6(6.38%)	25(26.60%)	12(12.77%)		
G2	43	6(6.38%)	25(26.60%)	12(12.77%)		
G3	8	1(1.06%)	7(7.45%)	0(0%)		
**T, n (%)**					3.7	0.7172
T1	84	13(13.54%)	47(48.96%)	24(25.00%)		
T2	6	0(0%)	5(5.21%)	1(1.04%)		
T3	5	0(0%)	3(3.13%)	2(2.08%)		
T4	1	0(0%)	1(1.04%)	0(0%)		
**N, n (%)**					4.736	0.3154
N0	45	5(10.00%)	36(72.00%)	4(8.00%)		
N1	3	0(0%)	2(4.00%)	1(2.00%)		
N2	2	0(0%)	1(2.00%)	1(2.00%)		
**M, n (%)**					3.462	0.4837
M0	46	5(10.00%)	36(72.00%)	5(10.00%)		
M1	4	0(0%)	3(6.00%)	1(2.00%)		
**FIGO stage, n (%)**					3.63	0.7267
I	37	5(10.00%)	29(58.00%)	3(6.00%)		
II	5	0(0%)	4(8.00%)	1(2.00%)		
III	4	0(0%)	3(6.00%)	1(2.00%)		
IV	4	0(0%)	3(6.00%)	1(2.00%)		
**Menopause, n (%)**					8.639	**0.0133**
YES	42	2(4.00%)	34(68.00%)	6(12.00%)		
NO	8	3(6.00%)	5(10.00%)	0(0%)		
**HPV, n (%)**					4.118	0.1276
YES	7	1(0.98%)	6(5.88%)	0(0%)		
NO	95	12(11.76%)	48(47.06%)	35(34.31%)		
**Chemotherapy, n (%)**					0.7308	0.6939
YES	1	0(0%)	1(0.84%)	0(0%)		
NO	118	14(11.76%)	68(57.14%)	36(30.25%)		
**Radiotherapy, n (%)**					1.474	0.4785
YES	2	0(0%)	2(1.68%)	0(0%)		
NO	117	14(11.76%)	67(56.30%)	36(30.25%)		
**Hypertension, n (%)**					1.555	0.4595
YES	39	3(2.61%)	26(22.61%)	10(8.70%)		
NO	76	10(8.70%)	42(36.52%)	24(20.87%)		
**Diabetes, n (%)**					5.189	0.0747
YES	14	0(0%)	12(11.01%)	2(1.83%)		
NO	95	14(12.84%)	52(47.71%)	29(26.61%)		
**Surgery, n (%)**					5.455	0.0654
YES	48	4(3.36%)	34(28.57%)	10(8.40%)		
NO	71	10(8.40%)	35(29.41%)	26(21.85%)		
**Smoking, n (%)**					-	-
YES	0	0(0%)	0(0%)	0(0%)		
NO	109	14(12.84%)	64(58.72%)	31(28.44%)		
**Drinking, n (%)**					-	-
YES	0	0(0%)	0(0%)	0(0%)		
NO	119	14(11.76%)	69(57.98%)	36(30.25%)		
**ER, n (%)**					3.05	0.5495
Negative	2	0(0%)	2(2.47%)	0(0%)		
Low positive	71	7(8.64%)	51(62.96%)	13(16.05%)		
High positive	8	0(0%)	5(6.17%)	3(3.70%)		
**PR, n (%)**					3.829	0.6998
-	4	0(0%)	3(3.23%)	1(1.08%)		
+	82	7(7.53%)	53(56.99%)	22(23.66%)		
+ +	6	1(1.08%)	4(4.30%)	1(1.08%)		
+ + +	1	0(0%)	0(0%)	1(1.08%)		
**Vimentin, n (%)**					8.1	0.2309
-	5	1(1.09%)	1(1.09%)	3(3.26%)		
+	80	8(8.70)	50(54.35%)	22(23.91%)		
+ +	4	0(0%)	4(4.35%)	0(0%)		
+ + +	3	0(0%)	3(3.26%)	0(0%)		
**p53, n (%)**					2.446	0.6543
-	7	0(0%)	6(6.52%)	1(1.09%)		
+	81	9(9.78%)	49(53.26%)	23(25.00%)		
+ +	0	0(0%)	0(0%)	0(0%)		
+ + +	4	0(0%)	3(3.26%)	1(1.09%)		

### G3BP1 is positively correlated with ERα in endometrial carcinoma

**Fig. 2:**
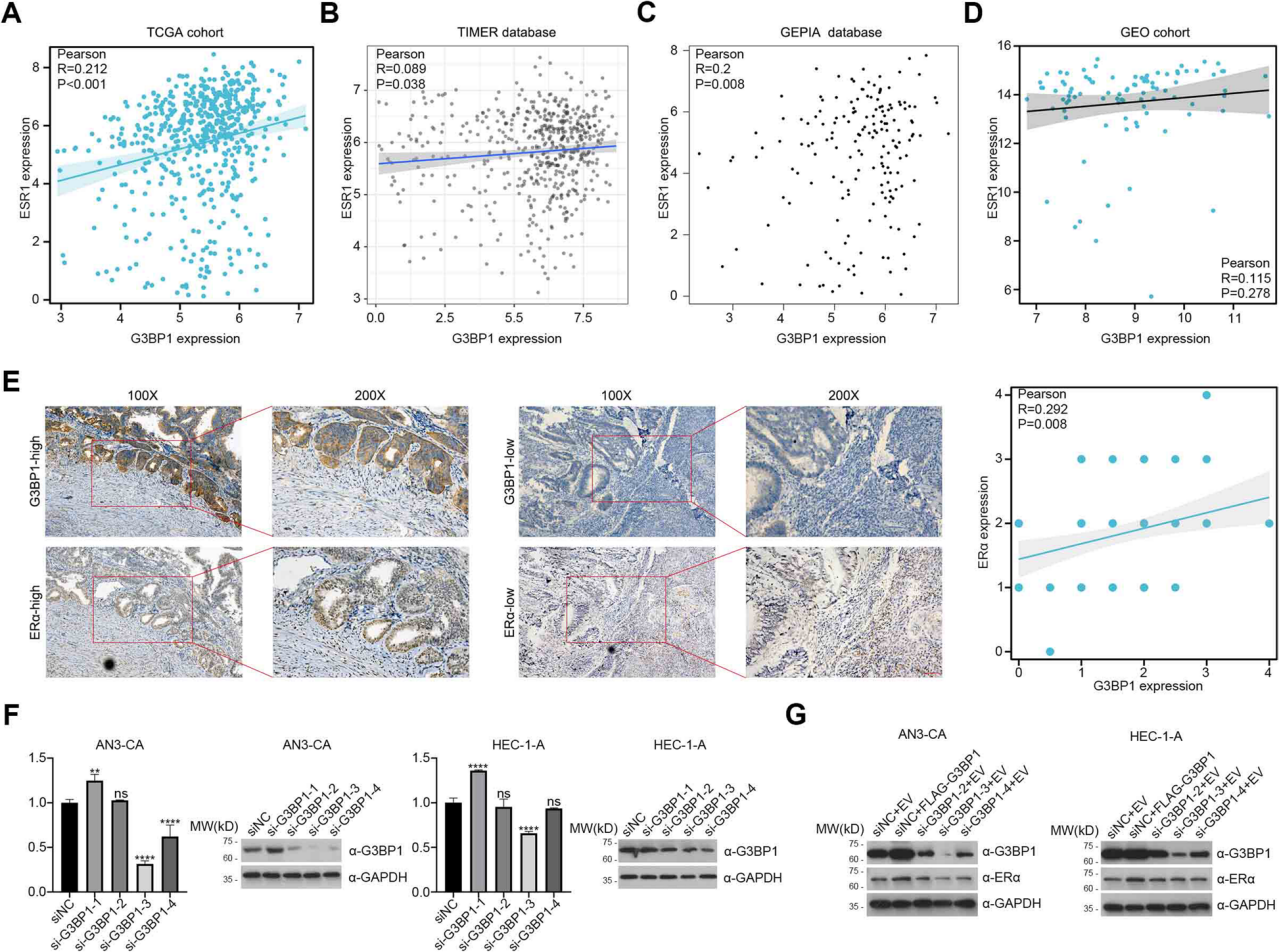
G3BP1 is positively correlated with ERα in endometrial carcinoma. **A** Correlation analysis between G3BP1 mRNA and ESR1 mRNA in EC based on the TCGA cohort (*n* = 552). **B**, **C** Correlation analysis between G3BP1 mRNA and ESR1 mRNA in EC based on the TCGA cohort (*n* = 552) using TIMER (*n* = 545) (**B**) and GEPIA (*n* = 552) (**C**) cohort. **D** Correlation analysis between G3BP1 mRNA and ESR1 mRNA in EC based on the GEO cohort (GSE17025) (*n* = 91). **E** Correlation analysis and representative images of G3BP1 protein and ERα in paraffin-embedded EC tissues by immunohistochemical staining (*n* = 120). **F** Four si-G3BP1 were transfected into HEC-1-A and AN3-CA cells. After 48 h, the mRNA and protein expression of G3BP1 were detected by qRT-PCR and western blot, respectively. **G** FLAG-G3BP1 and si-G3BP1 were co-transfected into HEC-1-A and AN3-CA cells. After 48 h of transfection, the cell lysates were prepared and the protein levels of G3BP1 and ERα were determined by western blot. Data are shown as mean ± SD (*n* = 3). *P < 0.05, **P < 0.01, ***P < 0.001, ****P < 0.0001

Although G3BP1 is highly expressed in EC, the underlying mechanism through which G3BP1 promotes the occurrence and development of EC remains to be studied. Given that ERα is the most widely studied biomarker and the best predictor of endocrine therapy response in patients with EC, we explored the possibility that G3BP1 may promote the development of EC by regulating the stability of ERα. To verify this notion, correlation analysis was performed on 552 EC samples from the TCGA cohort, and the results showed a positive correlation between G3BP1 and ESR1 at the mRNA level (Fig. 2A). Correlation analysis of EC samples in the TCGA cohort using TIMER (Fig. 2B) and GEPIA (Fig. 2C) cohort showed consistent results. However, based on 91 EC samples in the GEO cohort (GSE17025), there was no significant positive association between G3BP1 and ESR1 (Fig. 2D). To further explore the correlation between G3BP1 and ERα at the protein level, 120 pairs of paraffin-embedded EC samples were sequentially stained with G3BP1 and ERα. The results showed that G3BP1 and ERα were positively correlated at the protein level (Fig. 2E). Though western blot and qRT-PCR, we found that si-G3BP1-3 had the best knockdown effects at the protein and mRNA levels, respectively (Fig. 2F). We further tried to explore the role of G3BP1 in HEC-1-A and AN3-CA cells by overexpression or knockdown of *G3BP1*. The protein level of ERα in HEC-1-A and AN3-CA cells was positively changed with G3BP1 (Fig. 2G). Given that si-G3BP1-3 has the best knockdown effect on G3BP1 both at the protein and mRNA levels, si-G3BP1-3 was selected for subsequent studies.

### G3BP1 and G3BP1 Q392* competitively inhibits SPOP-mediated ubiquitination and degradation of ERα

**Fig. 3:**
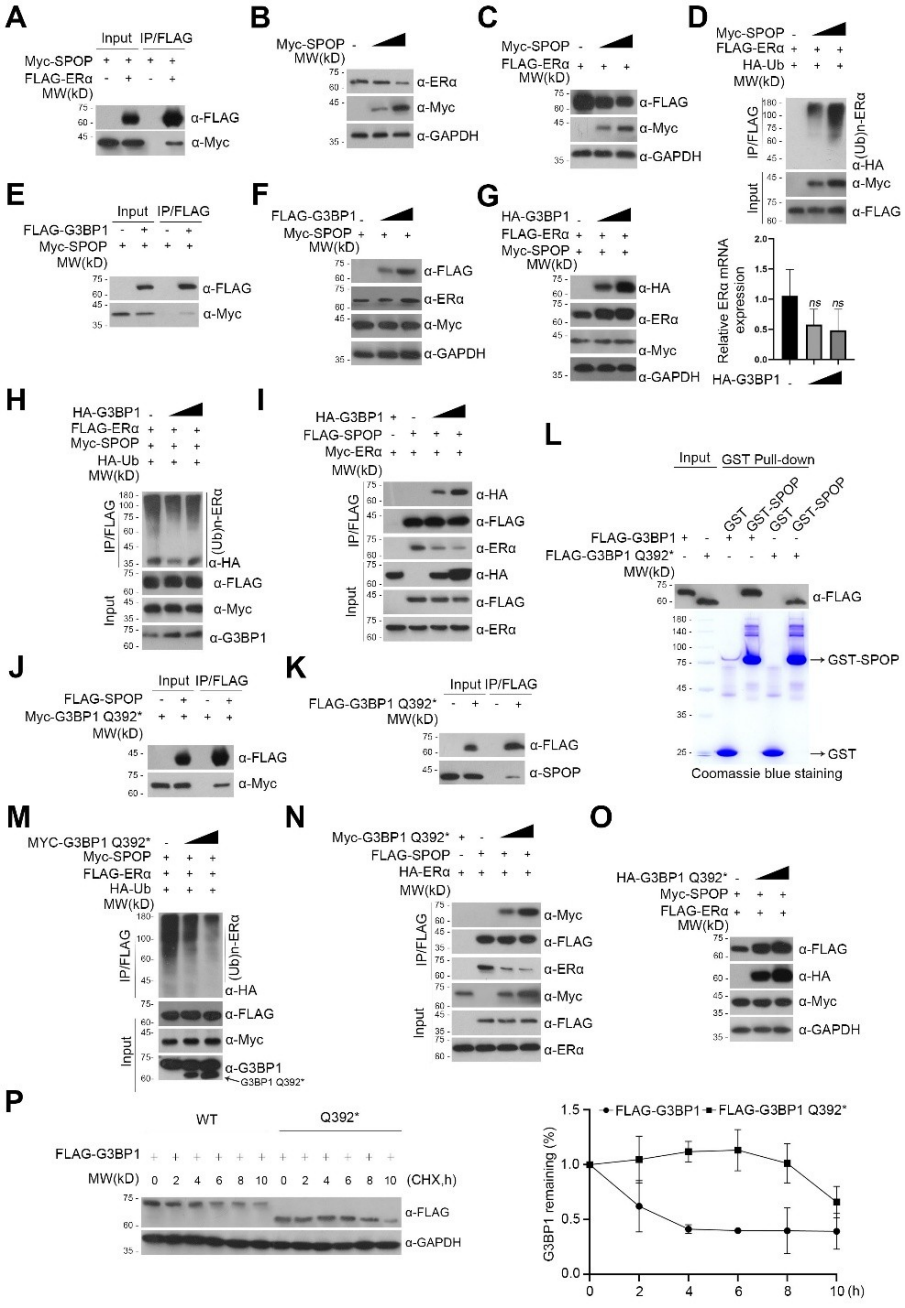
G3BP1 and G3BP1 Q392* competitively inhibits SPOP-mediated ubiquitination and degradation of ERα. **A** HEK293T cells were co-transfected with the indicator plasmids and treated with MG-132 (25 μM) for 8 h prior to lysis. 48 h after transfection, cell lysates were subjected to immunoprecipitation with anti-FLAG antibody and immunoblotted with anti-FLAG and anti-Myc antibodies. **B**, **C** HEK293T cells were transfected with the indicator plasmids. 48 h after transfection, cell lysates were prepared and the protein levels of ERα and SPOP were determined by western blot. **D**, **E** HEK 293 T cells were transfected with the indicated plasmids and treated with MG-132 (25 μM) for 8 h prior to lysis. 48 h after transfection, cell lysates were subjected to immunoprecipitation (IP) with anti-FLAG antibody and immunoblotted with indicator antibodies. **F**, **G** HEK293T cells were transfected with the indicator plasmids. 48 h after transfection, cell lysates were prepared. The protein and mRNA levels were determined by western blot and qRT-PCR, respectively. **H–K** HEK293T cells were co-transfected with the indicator plasmids. 48 h after transfection, cell lysates were subjected to immunoprecipitation with anti-FLAG antibody and immunoblotted with indicator antibodies. **L** Bacterially expressed GST-SPOP or GST bound glutathione-Sepharose beads and incubated with bacterially expressed FLAG-G3BP1 or FLAG-G3BP1 Q392*. Bound FLAG-G3BP1 or FLAG-G3BP1 Q392* was detected by Western blot with anti-FLAG antibody. GST and GST-SPOP were detected by western blot and Coomassie blue staining. **M**, **N** HEK293T cells were co-transfected with the indicator plasmids. 48 h after transfection, cell lysates were subjected to immunoprecipitation with anti-FLAG antibody and immunoblotted with indicator antibodies. **O** HEK293T cells were transfected with the indicator plasmids. 48 h after transfection, cell lysates were prepared. The protein level was determined by western blot. **P** HEK293T cells were transfected with G3BP1 wide-type and G3BP1 Q392*. After 48 h, cells were chased with 50 μg/mL cycloheximide (CHX). At the indicated time points, cell lysates were prepared for WB analyzes. Data are shown as mean ± SD (*n* = 3). *P < 0.05, **P < 0.01, ***P < 0.001, ****P < 0.0001

Although G3BP1 can up-regulate the expression of ERα in EC, the underlying mechanism is unknown. G3BP1 has previously been reported to competitively inhibit SPOP-mediated ubiquitination and degradation of AR [7]. In addition, ERα, like AR, is one of the substrates of SPOP [10]. Herein, we confirmed the SPOP-mediated ER ubiquitination and degradation in our experiments (Fig. 3A-D). Therefore, we speculate that G3BP1 may also upregulates ERα through the mechanism like AR. To verify this notion, firstly, we confirmed that G3BP1 is an interacting protein of SPOP by co-immunoprecipitation (Fig. 3E). In addition, by transient transfection of G3BP1, SPOP and ERα constructs, we found that G3BP1 inhibited SPOP-mediated ERα protein degradation without affecting the expression level of ERα mRNA (Fig. 3F, G). In addition, with the increasing concentration of G3BP1, the ubiquitination degree of ERα showed a gradual decline, which further confirmed that G3BP1 can increase the content of ERα in vivo by inhibiting SPOP-mediated ubiquitination and degradation of ERα mediated (Fig. 3H). Furthermore, with the increase expression of G3BP1, the binding of SPOP to G3BP1 was enhanced, while the binding of SPOP to ERα was weakened (F ig. 3I). This suggests that G3BP1 is a competitive inhibitor of SPOP, which can competitively inhibit the SPOP-mediated ubiquitination and degradation of ERα. As for G3BP1 Q392*, it has similar functions as wide-type G3BP1. Co-immunoprecipitation and western blot analysis in HEK293T cells showed that G3BP1 Q392* interacts with SPOP (Fig. 3J, K), and is further confirmed by GST pull-down assays in vitro (Fig. 3L). What is more, G3BP1 Q392* can also competitively inhibit the SPOP-meditated ubiquitination and degradation of Erα (Fig. 3M-O). Therefore, G3BP1 Q392* can also increase the expression of Erα. We further found that the half-life of G3BP1 Q392* is much longer than that of G3BP1 (Fig. 3P), suggesting it may play a more violent role in the occurrence and development of EC.

### The promoting effects of G3BP1 and G3BP1 Q392* on the proliferation and metastasis of endometrial carcinoma partly depend on SPOP/ERα axis

**Fig. 4:**
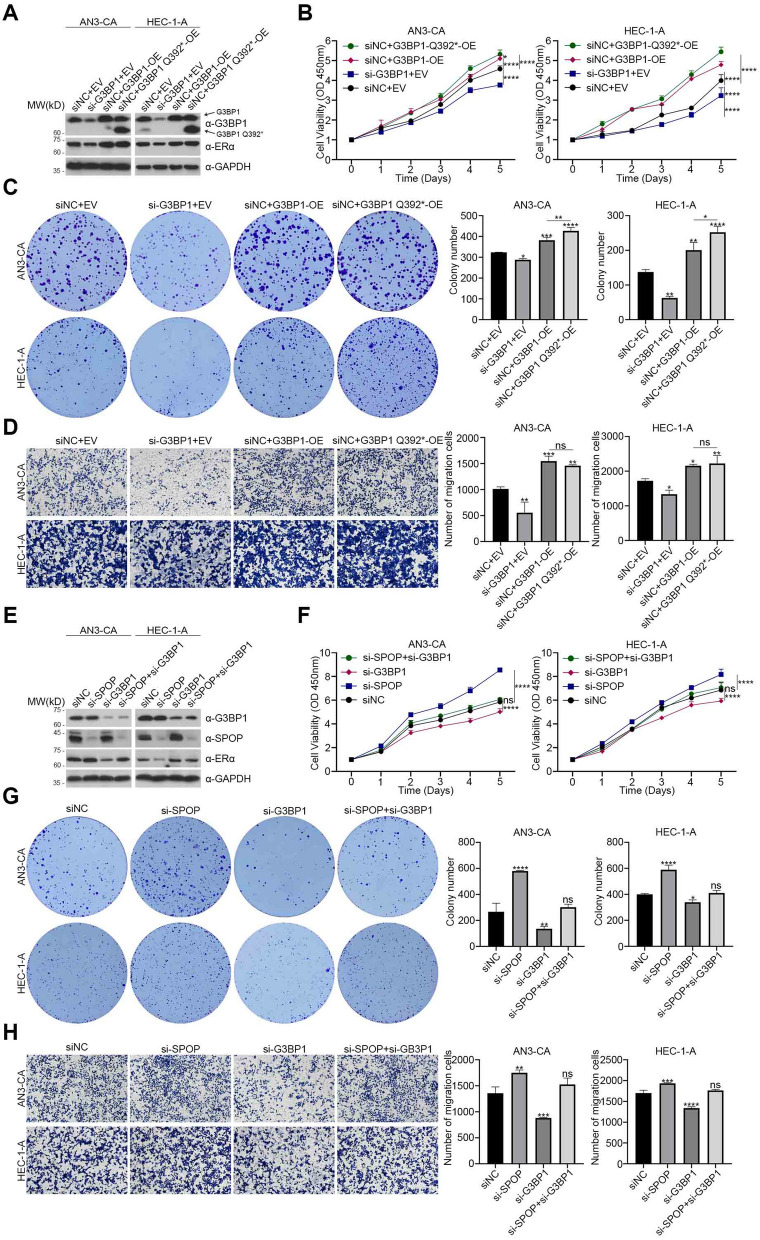
The effects of G3BP1 and G3BP1 Q392* on EC partly depend on SPOP/ERα axis. **A** AN3-CA and HEC-1-A cells were transfected with the indicator plasmids and si-G3BP1. 48 h after transfection, cell lysates were prepared and the protein levels of ERα and G3BP1 were determined by western blot. **B** CCK8 cell proliferation analysis was used to detect the proliferation ability of AN3-CA and HEC-1-A cells with G3BP1 knockdown, G3BP1 overexpression and G3BP1 Q392* overexpression. **C** Colony formation assay was used to detect the colony formation ability of AN3-CA and HEC-1-A cells with G3BP1 knockdown, G3BP1 overexpression and G3BP1 Q392* overexpression. **D** Cell migration assay was used to detect the metastasis ability of AN3-CA and HEC-1-A cells with G3BP1 knockdown, G3BP1 overexpression and G3BP1 Q392* overexpression. **E** AN3-CA and HEC-1-A cells were transfected with si-SPOP, si-G3BP1 and si-SPOP + si-G3BP1. 48 h after transfection, cell lysates were prepared and the protein levels of ERα, SPOP and G3BP1 were determined by western blot. **F** CCK8 cell proliferation analysis was used to detect the proliferation ability of AN3-CA and HEC-1-A cells with G3BP1 knockdown, SPOP knockdown and G3BP1 + SPOP knockdown. **G** Colony formation assay was used to detect the colony formation ability of AN3-CA and HEC-1-A cells with G3BP1 knockdown, SPOP knockdown and G3BP1 + SPOP knockdown. **H** Cell migration assay was used to detect the metastasis ability of AN3-CA and HEC-1-A cells with G3BP1 knockdown, SPOP knockdown and G3BP1 + SPOP knockdown. Data are shown as mean ± SD (*n* = 3). *P < 0.05, **P < 0.01, ***P < 0.001, ****P < 0.0001

To study the occurrence and development of EC induced by G3BP1 and G3BP1 Q392*, we established AN3-CA and HEC-1-A cells with stable expression of G3BP1, G3BP1 Q392*, and AN3-CA and HEC-1-A cells with stable knockdown of *G3BP1* (Fig. 4A). CCK8 cell proliferation analysis and colony formation assay showed that overexpression of G3BP1 dramatically promoted proliferation (Fig. 4B) and colony formation (Fig. 4C) of AN3-CA and HEC-1-A cells, while the abilities of the colony formation and proliferation were significantly inhibited in *G3BP1* knockdown cells (Fig. 4B, C). It is worth noting that G3BP1 Q392* is indeed better than G3BP1 in these assays (Fig. 4B, C). In addition, overexpression of G3BP1 and G3BP1 Q392* also promotes metastasis of AN3-CA and HEC-1-A cells, and vice versa (Fig. 4D). However, G3BP1 Q392* was not superior to G3BP1 in promoting metastasis (Fig. 4D). On the contrary, in CCK8 cell proliferation analysis and colony formation assay, the promoting effect of G3BP1 Q392* was much stronger than that of G3BP1.

Given that G3BP1 and G3BP1 Q392* are upstream inhibitors of SPOP, we further investigated whether this promoting effects on the proliferation and metastasis of EC partly depend on SPOP/ERα axis. AN3-CA and HEC-1-A cells with SPOP knockdown, G3BP1 knockdown and SPOP and G3BP1 co-knockdown were obtained by transient transfection of si-SPOP, si-G3BP1 and siSPOP plus si-G3BP1, respectively (Fig. 4E). Through CCK8 cell proliferation analysis, we found that SPOP knockdown alone could promote the proliferation of EC, while G3BP1 knockdown could suppress the proliferation of EC, and this suppressive effect could be partly reversed by the SPOP knockdown (Fig. 4F). Similar results were found in the colony formation capacity (Fig. 4G) and metastasis capacity (Fig. 4H). Moreover, the expression of ERα detected by western blot also changed correspondingly (Fig. 4E).

After G3BP1 or G3BP1 Q392* being overexpressed in *SPOP*-knockdown cells, we found that the cells with G3BP1 or G3BP1 Q392* overexpression combined with *SPOP* knockdown had greater proliferation and metastasis capacity than those with *SPOP*-knockdown alone (Fig. S2A-D). This may be due to *SPOP* knockdown only blocking the G3BP1/SPOP/ERα axis, but G3BP1 also has many other carcinogenic pathways, such as stress granule pathway. It is noteworthy that G3BP1 overexpression combined with *SPOP* knockdown has a stronger oncogenic effect than G3BP1 Q392* overexpression combined with *SPOP* knockdown (Fig. S2A-D). This evidence suggests that the oncogenic effect of G3BP1 Q392* on EC is more dependent on SPOP/ERα axis than G3BP1. In conclusion, the promoting effects of G3BP1 and G3BP1 Q392* on the proliferation and metastasis of EC are partly depended on SPOP/ERα axis.

### Fulvestrant can reverse the promoting effects of G3BP1 and G3BP1 Q392* on endometrial carcinoma

Fulvestrant is the only selective estrogen receptor degrader (SERD) approved by the Food and Drug Administration (FDA), which exhibits its antitumor effect through two mechanisms [2]. On the one hand, it competitively binds to ERα with estrogen, thereby inhibiting the activation of the ERα-mediated signaling pathways. On the other hand, it induces ERα degradation through UPS, thus inhibiting the development of cancers. Given that G3BP1 and G3BP1 Q392* promotes ERα accumulation via the SPOP/ERα axis, which leads to the proliferation and metastasis of EC, fulvestrant may reverse this effect.

**Fig. 5:**
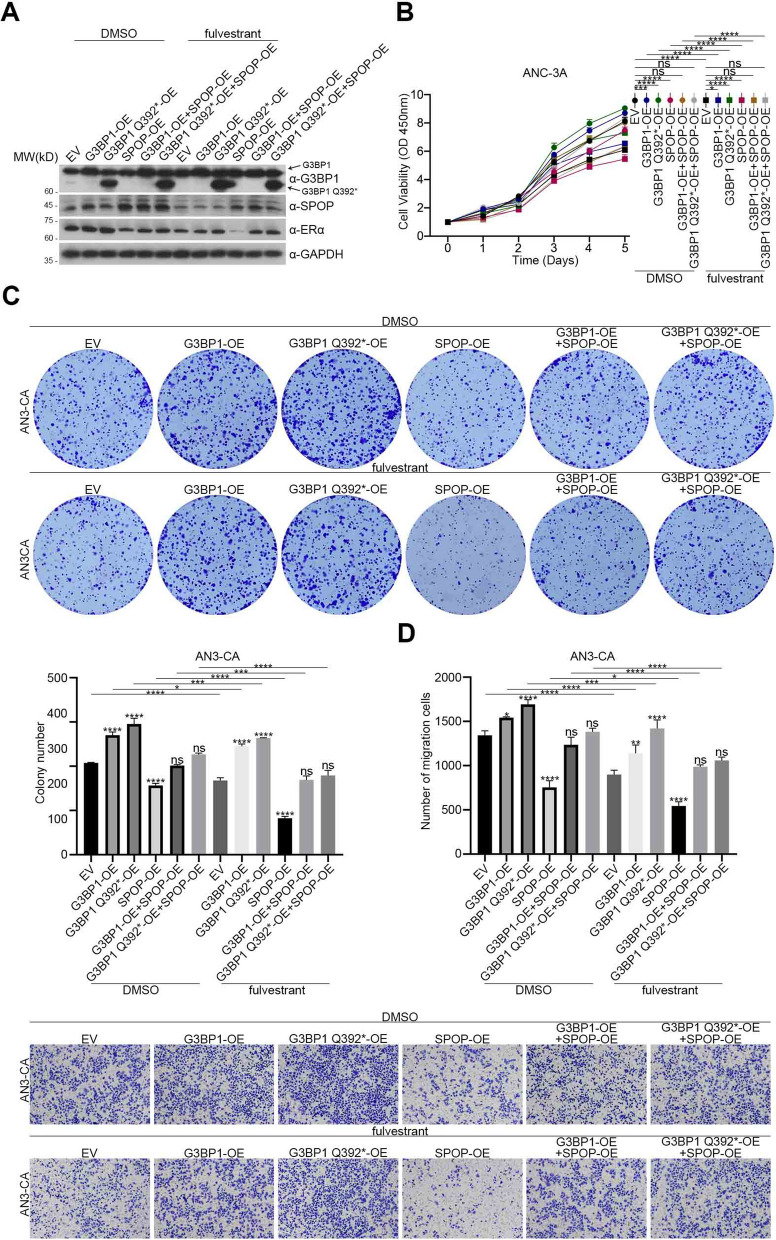
Fulvestrant can reverse the promoting effects of G3BP1 and G3BP1 Q392* on endometrial carcinoma. **A** AN3-CA cells were transfected with indicator plasmids. The cells were cultured with DMSO and fulvestrant (100 nM) in complete medium. 48 h after transfection, cell lysates were prepared and the protein levels of ERα, SPOP and G3BP1 were determined by western blot. **B** CCK8 cell proliferation analysis was used to detect the proliferation ability of AN3-CA cells. (**C**) Colony formation assay was used to detect the colony formation ability of AN3-CA cells. **D** Cell migration assay was used to detect the metastasis ability of AN3-CA cells. Data are shown as mean ± SD (*n* = 3). *P < 0.05, **P < 0.01, ***P < 0.001, ****P < 0.0001

To test this notion, we utilize DMSO and fulvestrant (100 nM) to treat the AN3-CA and HEC-1-A cells with G3BP1 overexpression, G3BP1 Q392* overexpression, SPOP overexpression, G3BP1 and SPOP co-overexpression, and G3BP1 Q392* and SPOP co-overexpression, respectively (Fig. 5A, S2A). The CCK8 analysis showed that G3BP1 overexpression or G3BP1 Q392* overexpression could promote the proliferation of EC cells in the DMSO-treated group, and the effect of G3BP1 Q392* was stronger. When SPOP was overexpressed in G3BP1/G3BP1 Q392* overexpression cells, the proliferation capacity of EC cells was reversed (Fig. 5B, S2B) and the expression of ERα protein was also changed correspondingly (Fig. 5A, S2A), indicating that G3BP1/G3BP1 Q392* promoted the proliferation of EC through the SPOP/ERα axis. Furthermore, compared with DMSO-treated EC cells, the proliferative ability of G3BP1/G3BP1 Q392* overexpression treated with fulvestrant was reduced (Fig. 5B, S2B), suggesting that fulvestrant could reverse the proliferative effect of G3BP1/G3BP1 Q392*. In addition, the expression level of ERα in EC cells treated with fulvestrant was lower than that with DMSO (Fig. 5A, S2A), suggesting that fulvestrant reverses the proliferative effect of G3BP1/G3BP1 Q392* through the G3BP1/SPOP/ERα axis. Similar results were showed in the colony formation capacity (Fig. 5C, S2C) and metastasis capacity (Fig. 5D, S2D). It is concluded that fulvestrant can reverse the proliferation and metastasis of EC mediated by G3BP1/G3BP1 Q392*.

**Fig. 6:**
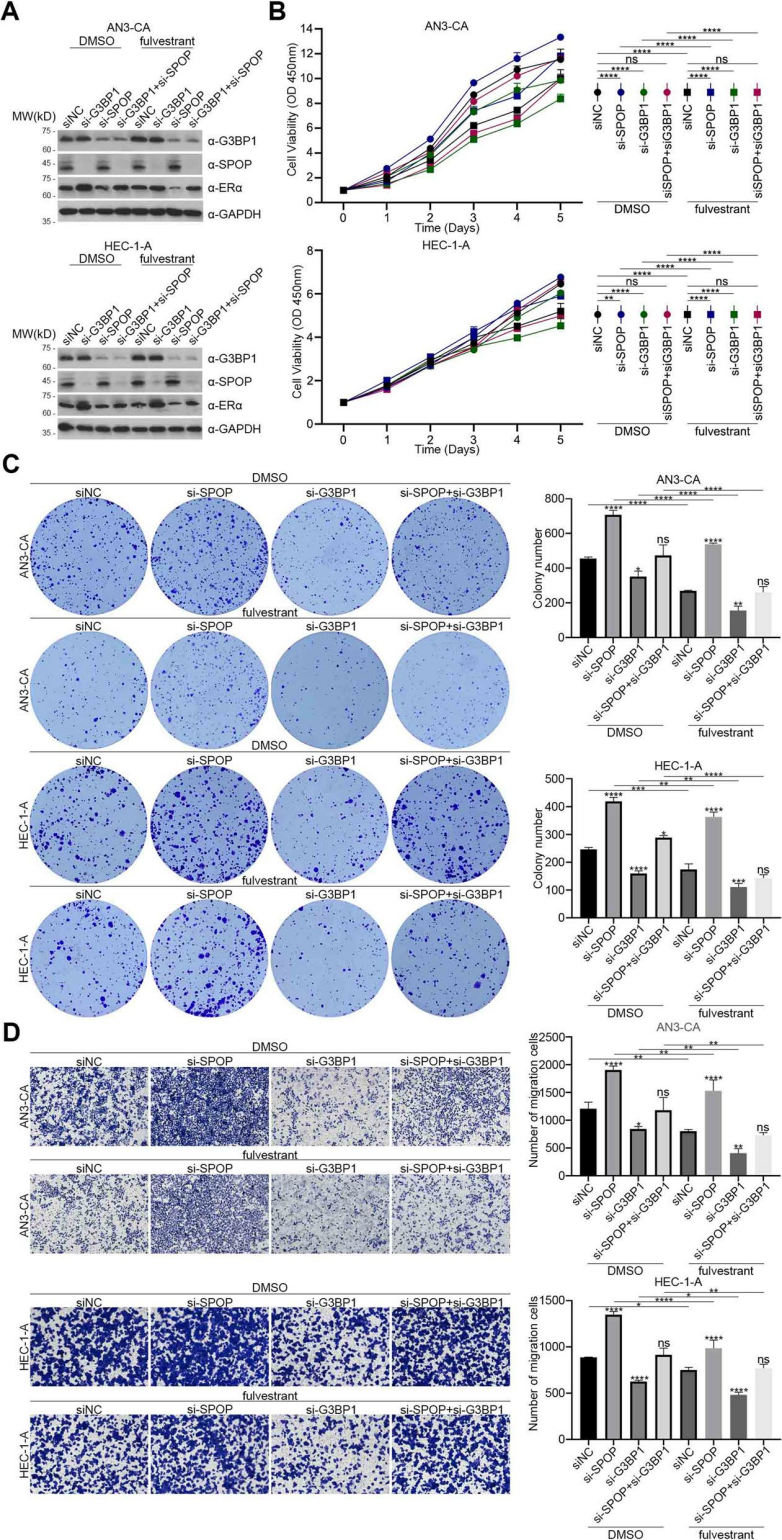
Fulvestrant combined with G3BP1 knockdown could improve the efficacy of fulvestrant. **A** AN3-CA and HEC-1-A cells were transfected with the indicator si-RNA. The cells were cultured with DMSO and fulvestrant (100 nM) in complete medium. 48 h after transfection, cell lysates were prepared and the protein levels of ERα, SPOP and G3BP1 were determined by western blot. **B** CCK8 cell proliferation analysis was used to detect the proliferation ability of AN3-CA and HEC-1-A cells. **C** Colony formation assay was used to detect the colony formation ability of AN3-CA and HEC-1-A cells. **D** Cell migration assay was used to detect the metastasis ability of AN3-CA and HEC-1-A cells. Data are shown as mean ± SD (*n* = 3). *P < 0.05, **P < 0.01, ***P < 0.001, ****P < 0.0001

Furthermore, we utilize DMSO and fulvestrant (100 nM) to treat the AN3-CA and HEC-1-A cells with SPOP knockdown, G3BP1 knockdown and SPOP and G3BP1 co-knockdown (Fig. 6A). CCK8 cell proliferation analysis showed that G3BP1 knockdown inhibited the proliferation of EC (Fig. 6B). Combined with SPOP knockdown, this effect was reversed (Fig. 6B), and the expression of ERα was changed correspondingly (Fig. 6A). All of these suggest that G3BP1 promotes the proliferation of EC cells through SPOP/ERα axis. In addition, compared with DMSO-treated EC cells, the proliferation capacity of G3BP1 knockdown EC cells treated with fulvestrant was the lowest (Fig. 6B), indicating that fulvestrant combined with G3BP1 knockdown improved the efficacy of fulvestrant. Similarly, the colony formation (Fig. 6C) and metastasis (Fig. 6D) capacity of EC showed the same results. Thus, fulvestrant combined with G3BP1 knockdown could improve the inhibitory effect of fulvestrant on the proliferation and metastasis of EC.

Therefore, it can be inferred that in EC patients with high G3BP1, fulvestrant can be applied to reverse the occurrence and development of EC. In addition, ERα expression is often higher in patients with high G3BP1, so G3BP1 can improve the sensitivity of patients with EC to fulvestrant. Since G3BP1 Q392* has a stronger oncogenic effect than G3BP1, fulvestrant may be more effective in EC patients with G3BP1 Q392*.

## Discussion

In this article, we identified a carcinogenic protein G3BP1 in EC that is positively associated with ERα protein expression. It promotes ERα accumulation by inhibiting the SPOP-mediated ERα ubiquitination and degradation, which leads to the proliferation and metastasis of EC. In addition, G3BP1 Q392* displays more suppressive effect than wildtype G3BP1, due to its longer half-life than G3BP1, but these effects could be reversed by fulvestrant, providing a promising idea for improving the efficacy of fulvestrant in EC patients with G3BP1-positive plus ERα-positive.

Notably, the expression of G3BP1 differs only at the protein level, but not at the mRNA level, suggesting that some post-translational modifications of G3BP1may be disrupted, resulting in the high expression of G3BP1 protein in EC. However, there are few studies on the post-translational modification of G3BP1, which mainly focus on the methylation, phosphorylation, and acetylation. Moreover, these post-translational modifications only affected the G3BP1-mediated SGs assembly but did not affect the expression of G3BP1. For further study, KEGG analysis and GO analysis were performed on genes associated with overexpression of G3BP1 protein based on CPTAC cohort (Fig. S1D, E). KEGG analysis is enriched in a variety of human diseases (Fig. S1D), which may be attributed to the fact that G3BP1 protein can mediate viral infection via SGs. GO analysis suggested that these genes were mainly enriched in gene transcription and ribosome (Fig. S1E), which was consistent with the characteristics of SGs assembly. Thus, the ubiquitin proteasome system (UPS) that mediates the degradation of G3BP1 has never been studied. Therefore, future studies can search for specific E3 ligase mediating the ubiquitination and degradation of G3BP1 in EC cells by mass spectrometry or yeast two-hybrid technology.

Secondly, we only demonstrated the effects of G3BP1 and G3BP1 Q392* on EC in the cell model, and these effects could be reversed by fulvestrant. However, we lack further validation at the mouse models and patients-derived organoid models. Mouse models of conditionally knocked-in G3BP1 Q392* or overexpression of G3BP1 were able to further examine the effect of G3BP1 Q392* and G3BP1 on EC development. It is worth studying whether the tumor size and pathological grade of mice were reduced after the application of fulvestrant. At the organoid level, EC cells were extracted from EC patients with G3BP1 Q392*and cultured into organoids in vitro to test their therapeutic response to fulvestrant.

To date, resveratrol, epigallocatechin gal-late and GAP161 have all been shown to target and interfere with G3BP1, thereby acting as inhibitors during tumorigenesis [6]. Unfortunately, these drugs are not yet in clinical trials, only in the research phase. Among them, GAP161 can improve the sensitivity of colon cancer cells to cisplatin by down-regulating G3BP1 [12]. It is possible that GAP161, when combined with fulvestrant, can further improve the efficacy of fulvestrant against EC. In addition, anti-G3BP1 drugs may be considered in combination with other anti-ERα drugs. In clinical practice, there are two main classes of estrogen receptor antagonists, namely selective estrogen receptor modulators (SERMs) and SERDs [2]. The representative drugs of SERMs and SREDS are tamoxifen and fulvestrant respectively [3]. Anti-G3BP1 drugs combined with tamoxifen may also be a good option. However, studies have shown that patients have an increased risk of EC with the use of tamoxifen. Thus, tamoxifen in combination with G3BP1 antagonists can improve the efficacy of the drug to some extent, but whether this can counteract its own carcinogenic effect is unclear.

## Supplementary Information

**Additional file 1:** **Supplementary Table 1.** Primer for qRT-PCR and constructions, siRNA oligonucleotide sequences. **Supplementary Fig. 1.** Bioinformatics analysis of G3BP1 mRNA and protein. (A) Kaplan-Meier survival curves of EC based on the TCGA cohort. (B) Heat map of clinical characteristics of EC based on TCGA cohort. (C) Heat map of clinical characteristics of EC based on CPTAC cohort. (D) Gene co-expression circle of G3BP1 based on TCGA cohort. (E) KEGG analysis was performed based on CPTAC cohort. (F) Based on CPTAC cohort, R package was used to enrich gene ontology, including BP, molecular MF and CC. Data are shown as mean ± SD (*n* = 3). *P < 0.05, **P < 0.01, ***P < 0.001, ****P < 0.0001. **Supplementary Fig.** 2. The promoting effects of G3BP1 and G3BP1 Q392* on the proliferation and metastasis of endometrial carcinoma partly depend on SPOP/ERα axis. (A) AN3-CA and HEC-1-A cells were transfected with indicator plasmids. 48h after transfection, cell lysates were prepared and the protein levels of ERα, SPOP and G3BP1 were determined by western blot. (B) CCK8 cell proliferation analysis was used to detect the proliferation ability of AN3-CA and HEC-1-A cells. (C) Colony formation assay was used to detect the colony formation ability of AN3-CA and HEC-1-A cells. (D) Cell migration assay was used to detect the metastasis ability of AN3-CA and HEC-1-A cells. Data are shown as mean ± SD (*n* = 3). *P < 0.05, **P < 0.01, ***P < 0.001, ****P < 0.0001. **Supplementary Fig. 3.** Fulvestrant can reverse the promoting effects of G3BP1 and G3BP1 Q392* on endometrial carcinoma. (A) HEC-1-A cells were transfected with indicator plasmids. The cells were cultured with DMSO and fulvestrant (100nM) in complete medium. 48h after transfection, cell lysates were prepared and the protein levels of ERα, SPOP and G3BP1 were determined by western blot. (B) CCK8 cell proliferation analysis was used to detect the proliferation ability of HEC-1-A cells. (C) Colony formation assay was used to detect the colony formation ability of HEC-1-A cells. (D) Cell migration assay was used to detect the metastasis ability of HEC-1-A cells. Data are shown as mean ± SD (*n* = 3). *P < 0.05, **P < 0.01, ***P < 0.001, ****P < 0.0001

**Additional file 2.**

## Data Availability

The datasets used and/or analysed during the current study are available from the corresponding author on reasonable request.

## Abbreviations

AR: Androgen receptor
CRL: Cullin3-RING ubiquitin ligase
EC: Endometrial cancer
ERE: Estrogen response element
ERα: Estrogen receptor alpha
FDA: Food and Drug Administration
G3BP1: Ras-GTPase-activating protein binding protein 1
OS: Overall survival
PFS: Progression-free survival
SERD: Selective estrogen receptor degrader
SERM: Selective estrogen receptor modulator
SPOP: Speckle-type POZ protein
UPS: Ubiquitin proteasome system
