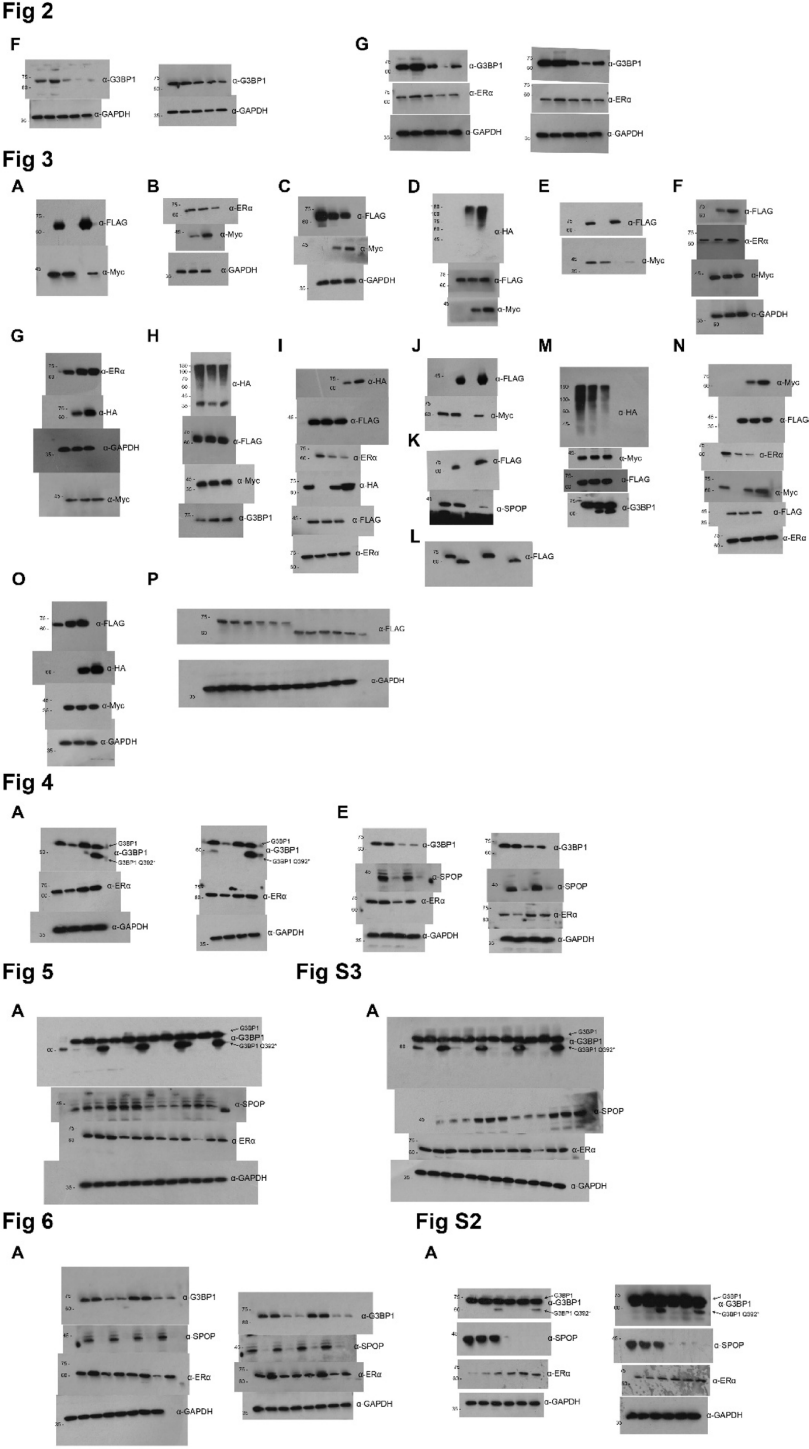# Correction: Endometrial cancer (EC) derived G3BP1 overexpression and mutant promote EC tumorigenesis and metastasis via SPOP/ERα axis

**DOI:** 10.1186/s12964-025-02505-4

**Published:** 2025-10-24

**Authors:** Yidong Ge, Jiabei Jin, Gun Chen, Jinyun Li, Meng Ye, Xiaofeng Jin

**Affiliations:** 1 Department of Radiotherapy and Chemotherapy, The First Hospital of Ningbo University, Ningbo University, Ningbo, 315010 China; 2 The Affiliated People’s Hospital of Ningbo University, Ningbo, 315040 China; 3 Department of Biochemistry and Molecular Biology, Zhejiang Key Laboratory of Pathophysiology, Health Science Center, Medical School of Ningbo University, Ningbo University, Ningbo, 315211 Zhejiang China

**Correction: Cell Commun Signal 21, 303 (2023)**

**https://doi.org/10.1186/s12964-023-01342-7**

Following publication of the original article [1], the authors identified errors in Fig. 3 and Supplementary Fig. 2.

1. In the Fig. 3O of the manuscript, the picture of WB strip of “GAPDH” were misused.
2. In the Supplementary Fig. 2 of the manuscript, the picture of Transwell figures of “NC + G3BP1-OE” and “NC + G3BP1 Q392*-OE” of AN3-CA were misused.

The incorrect Fig. 3 and Supplementary Fig. 2 is given below.

Misused data for Figure 3


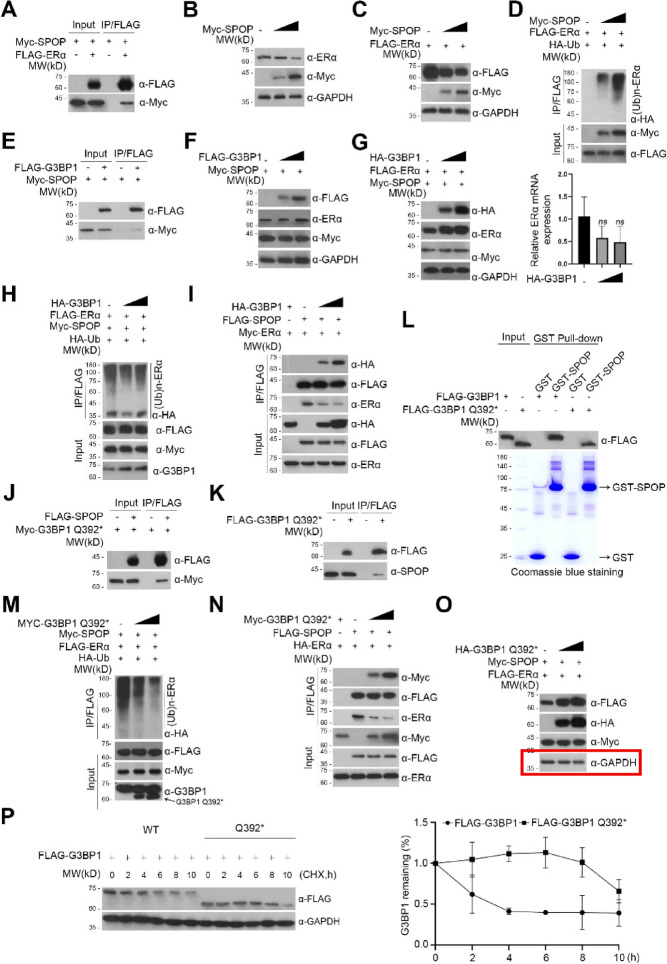


Misused data for Supplementary Figure 2


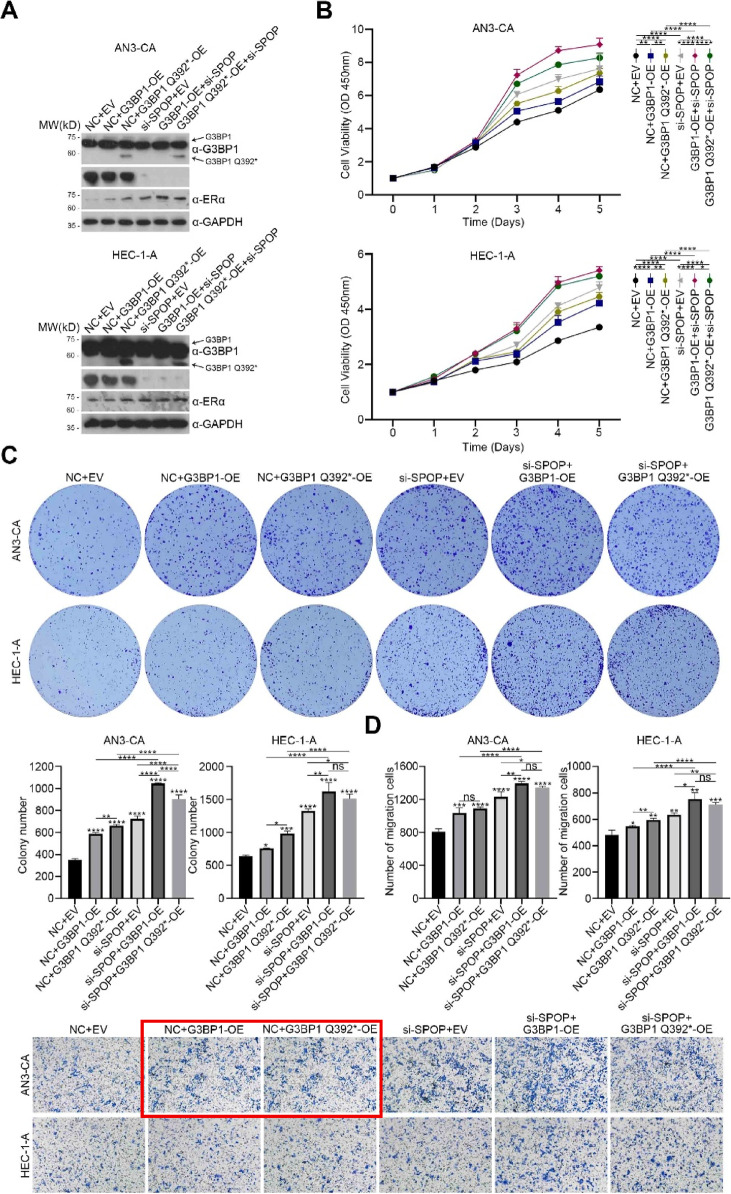


The correct Fig. 3, Supplementary Fig. 2 and Original Western Blots are given below.

New Figure 3


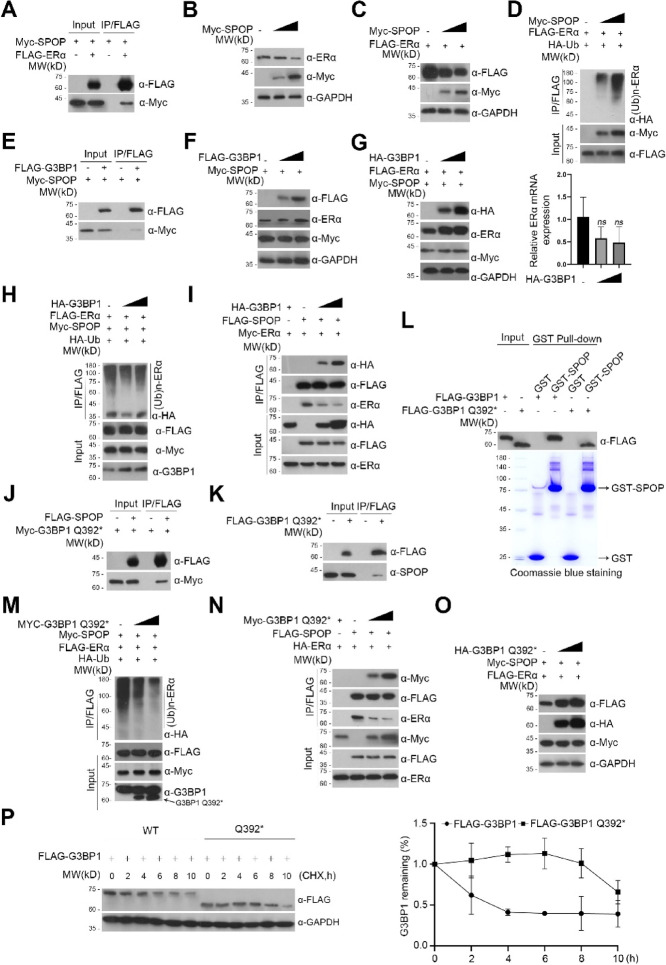


New Supplementary Figure 2


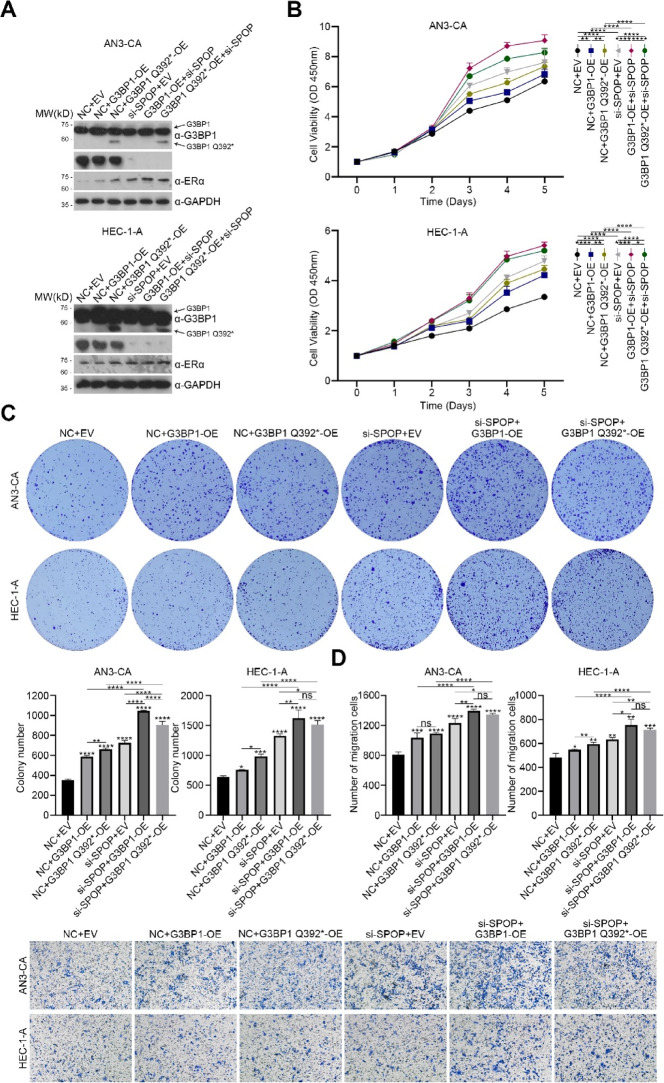


New Original Western Blots