# Supplementary material for: Endometrial cancer (EC) derived G3BP1 overexpression and mutant promote EC tumorigenesis and metastasis via SPOP/ERα axis
Source: Cell Commun Signal. 2023 Oct 30;21:303. doi: 10.1186/s12964-023-01342-7 (PMC10614411; doi:10.1186/s12964-023-01342-7)

**Supplementary Table 1: Primer for qRT-PCR and constructions, siRNA oligonucleotide sequences.**

| **SYBR®Green qRT-PCR primers** | |
| --- | --- |
| G3BP1 | RTF: 5’-AAGAGTGCGAGAACAACGAA-3’  RTR: 5’-TGGTGACTGTCAGGGTGTCT-3’ |
| ESR1 | RTF: 5’-GGCTACATCATCTCGGTTCC-3’  RTR: 5’-TCAGGGTGCTGGACAGAAA-3’ |
| GAPDH | RTF: 5’-CATGGCCTTCCGTGTTCCTA-3’  RTR: 5’-CCCTCAGATGCCTGCTTCA-3’ |
| **siRNA oligonucleotide sequences** | |
| si-G3BP1-1 | 5’-GAAAGAACUCUUCUUAUGU-3’ |
| si-G3BP1-2 | 5’-CAAGAUUCGCCAUGUUGAU-3’ |
| si-G3BP1-3 | 5’-GUAAUGACAUGGAAGAACA-3’ |
| si-G3BP1-4 | 5’-CAAAUCAGAGCUUAAAGAU-3’ |
| si-SPOP | 5’-GGAUGAUGUAAAUGAGCAA-3’ |
| **construction primers** | |
| ERα-EA | 5’-AAAGAATTCGGATGACCATGACCCTCCACACCAA-3’ |
| ERα-XB | 5’-AAACTCGAGTCAGACCGTGGCAGGGAAACCCTC-3’ |
| G3BP1-EA | 5’-AAAGAATTCGGATGGTGATGGAGAAGCCTAGTCC -3’ |
| G3BP1-NB | 5’-AAAGCGGCCGCTCACTGCCGTGGCGCAAGCCCCC3’ |
| G3BP1-Q68X-NB | 5’-AAAGCGGCCGCTCATGACATCACTTTCCTGTGGA-3’ |
| G3BP1-Q392X-NB | 5’-AAAGCGGCCGCTCAAACAGGCTCAGAATCATC-3’ |

**Supplementary Fig.1**


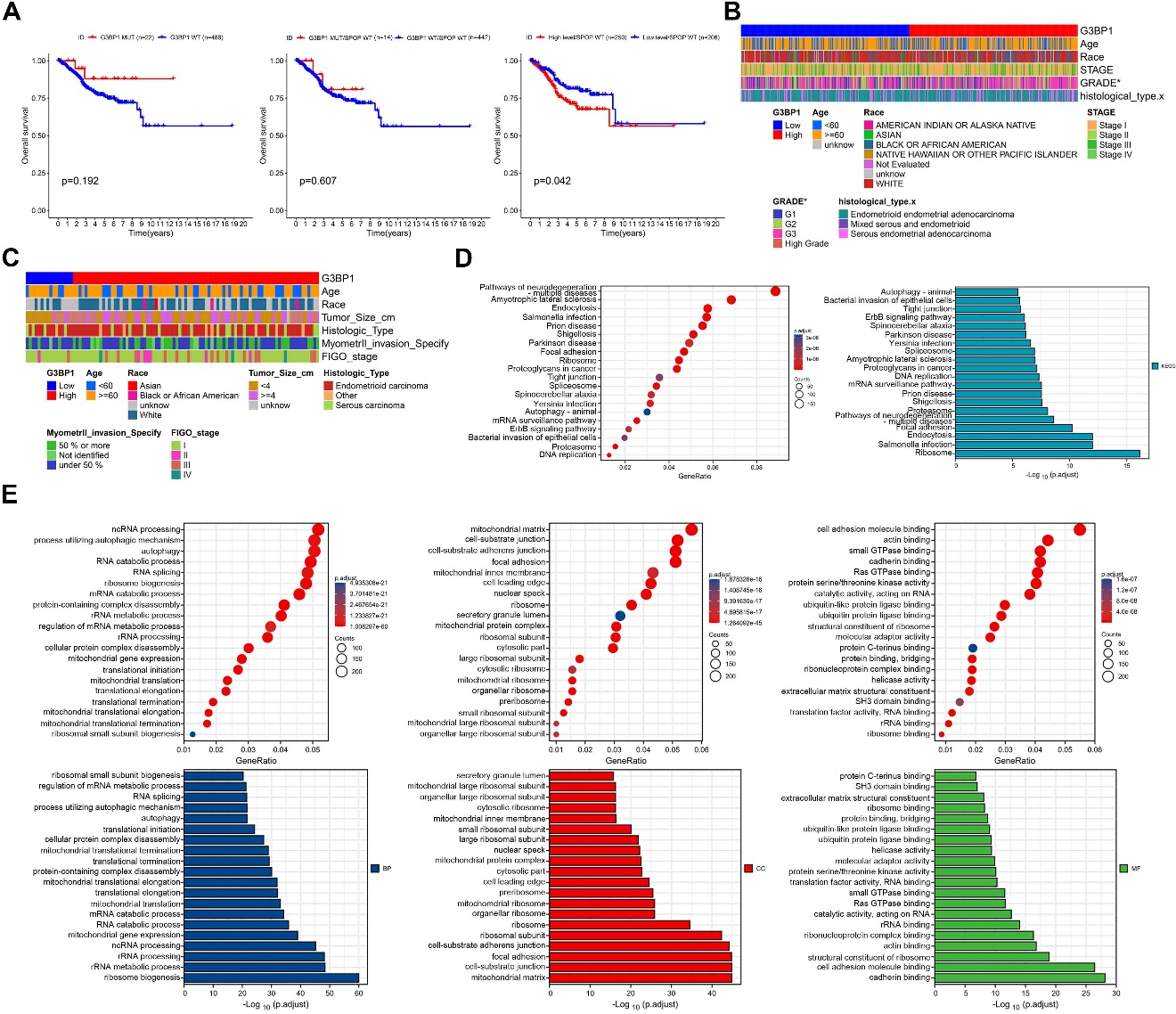


**Supplementary Fig.1 Bioinformatics analysis of G3BP1 mRNA and protein.** (**A**) Kaplan-Meier survival curves of EC based on the TCGA cohort. (**B**) Heat map of clinical characteristics of EC based on TCGA cohort. (**C**) Heat map of clinical characteristics of EC based on CPTAC cohort. (**D**) Gene co-expression circle of G3BP1 based on TCGA cohort. (**E**) KEGG analysis was performed based on CPTAC cohort. (**F**) Based on CPTAC cohort, R package was used to enrich gene ontology, including BP, molecular MF and CC. Data are shown as mean ± SD (n = 3). *P < 0.05, **P < 0.01, ***P < 0.001, ****P < 0.0001

**Supplementary Fig.2**


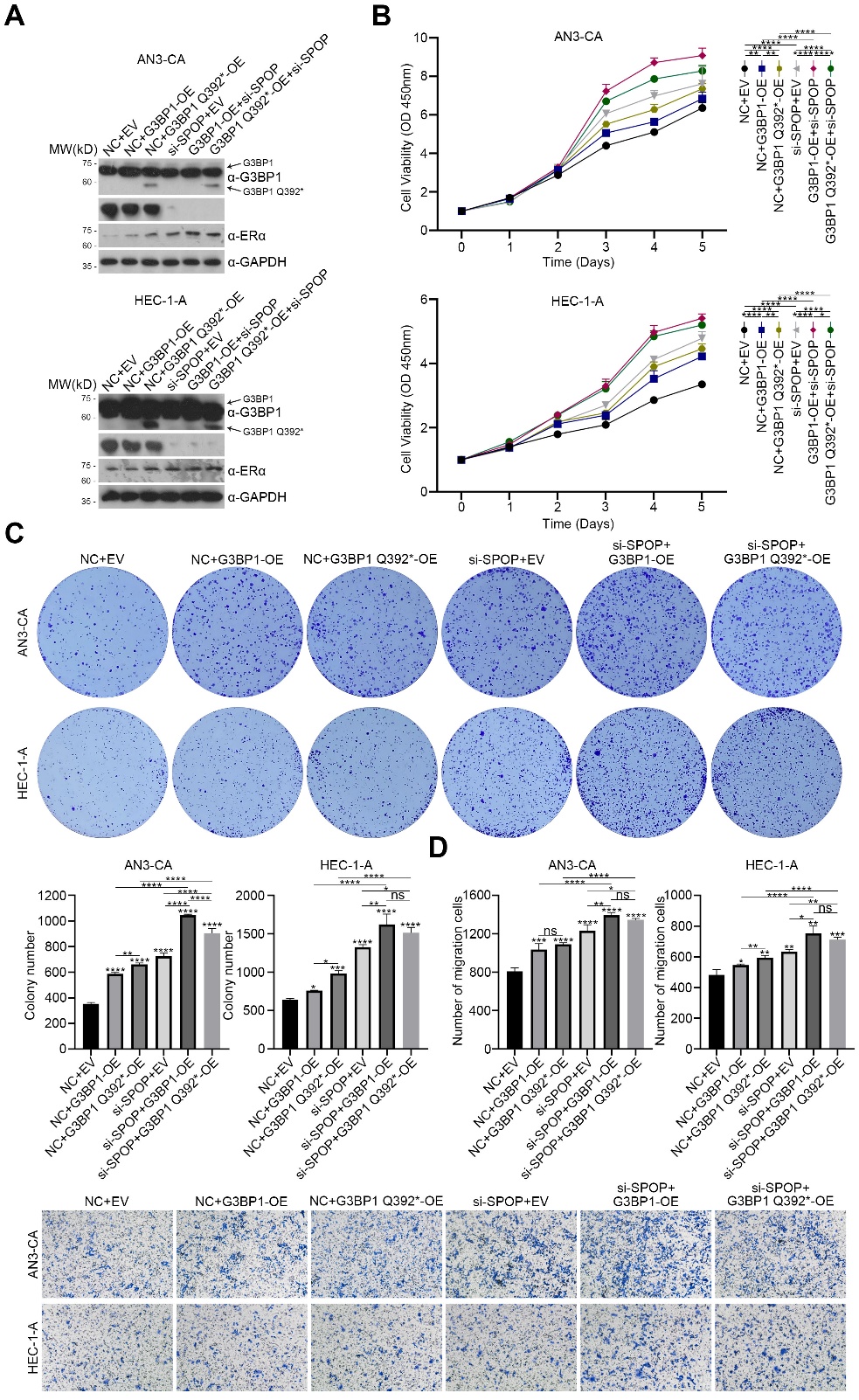


**Supplementary Fig.2 The promoting effects of G3BP1 and G3BP1 Q392* on the proliferation and metastasis of endometrial carcinoma partly depend on SPOP/ERα axis.** (**A**) AN3-CA and HEC-1-A cells were transfected with indicator plasmids. 48h after transfection, cell lysates were prepared and the protein levels of ERα, SPOP and G3BP1 were determined by western blot. (**B**) CCK8 cell proliferation analysis was used to detect the proliferation ability of AN3-CA and HEC-1-A cells. (**C**) Colony formation assay was used to detect the colony formation ability of AN3-CA and HEC-1-A cells. (**D**) Cell migration assay was used to detect the metastasis ability of AN3-CA and HEC-1-A cells. Data are shown as mean ± SD (n = 3). *P < 0.05, **P < 0.01, ***P < 0.001, ****P < 0.0001

**Supplementary Fig.3**


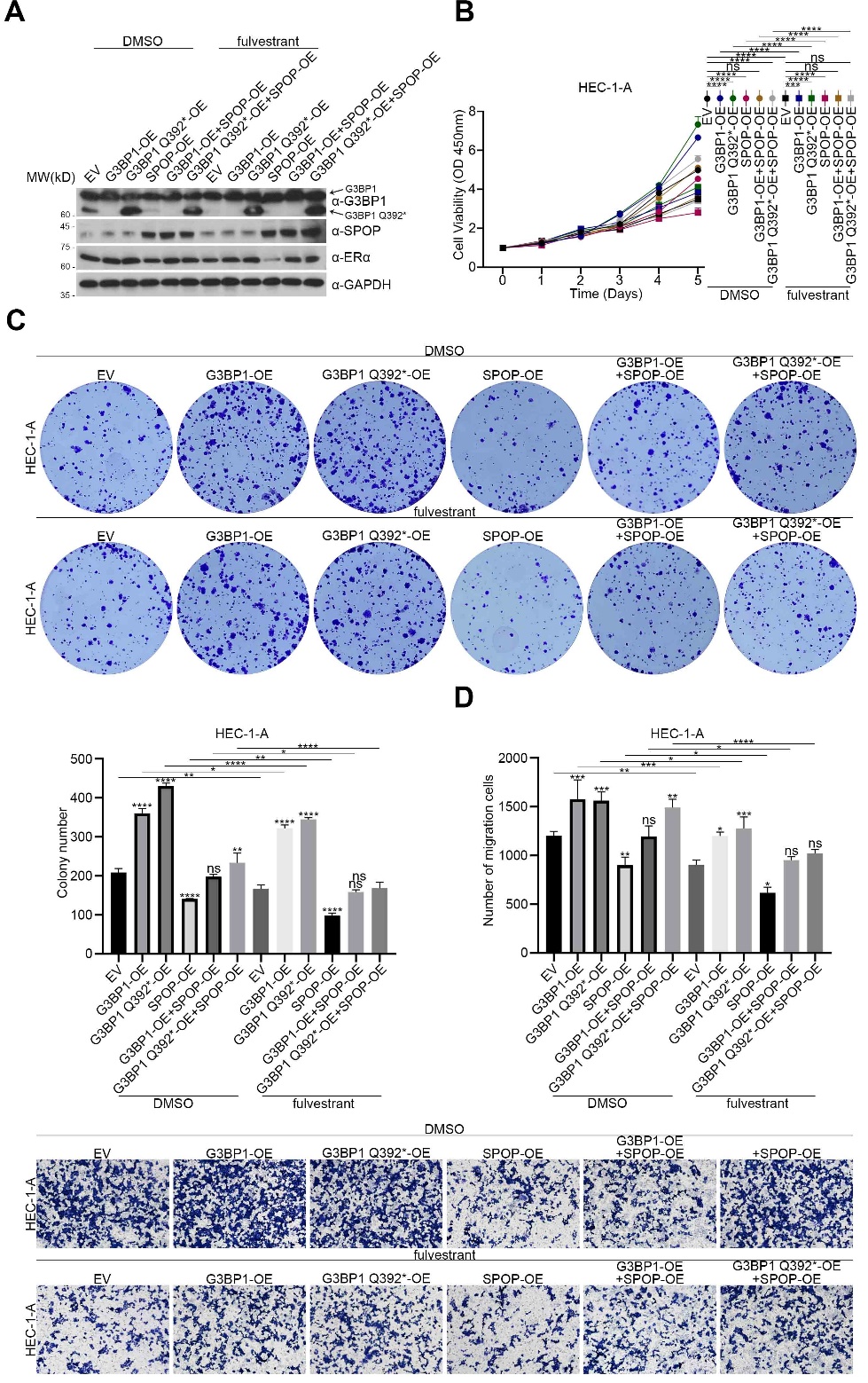


**Supplementary Fig.3 Fulvestrant can reverse the promoting effects of G3BP1 and G3BP1 Q392* on endometrial carcinoma.** (**A**) HEC-1-A cells were transfected with indicator plasmids. The cells were cultured with DMSO and fulvestrant (100nM) in complete medium. 48h after transfection, cell lysates were prepared and the protein levels of ERα, SPOP and G3BP1 were determined by western blot. (**B**) CCK8 cell proliferation analysis was used to detect the proliferation ability of HEC-1-A cells. (**C**) Colony formation assay was used to detect the colony formation ability of HEC-1-A cells. (**D**) Cell migration assay was used to detect the metastasis ability of HEC-1-A cells. Data are shown as mean ± SD (n = 3). *P < 0.05, **P < 0.01, ***P < 0.001, ****P < 0.0001

**Original Western Blots**


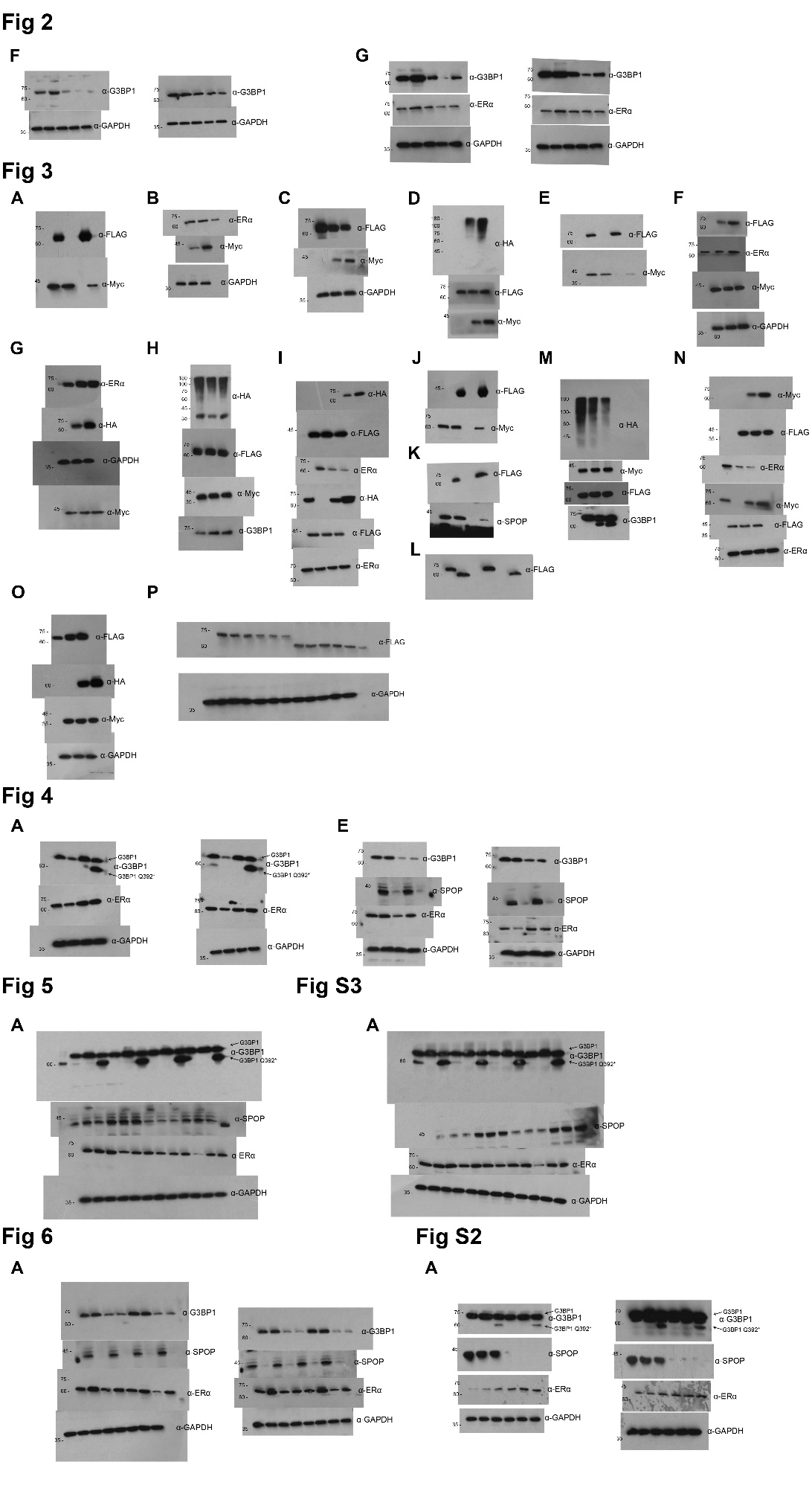

Supplement: Supplementary file 2 — Additional file 1: Supplementary Table 1. Primer for qRT-PCR and constructions, siRNA oligonucleotide sequences. Supplementary Fig. 1. Bioinformatics analysis of G3BP1 mRNA and protein. (A) Kaplan-Meier survival curves of EC based on the TCGA cohort. (B) Heat map of clinical characteristics of EC based on TCGA cohort. (C) Heat map of clinical characteristics of EC based on CPTAC cohort. (D) Gene co-expression circle of G3BP1 based on TCGA cohort. (E) KEGG analysis was performed based on CPTAC cohort. (F) Based on CPTAC cohort, R package was used to enrich gene ontology, including BP, molecular MF and CC. Data are shown as mean ± SD (n = 3). *P < 0.05, **P < 0.01, ***P < 0.001, ****P < 0.0001. Supplementary Fig. 2. The promoting effects of G3BP1 and G3BP1 Q392* on the proliferation and metastasis of endometrial carcinoma partly depend on SPOP/ERα axis. (A) AN3-CA and HEC-1-A cells were transfected with indicator plasmids. 48h after transfection, cell lysates were prepared and the protein levels of ERα, SPOP and G3BP1 were determined by western blot. (B) CCK8 cell proliferation analysis was used to detect the proliferation ability of AN3-CA and HEC-1-A cells. (C) Colony formation assay was used to detect the colony formation ability of AN3-CA and HEC-1-A cells. (D) Cell migration assay was used to detect the metastasis ability of AN3-CA and HEC-1-A cells. Data are shown as mean ± SD (n = 3). *P < 0.05, **P < 0.01, ***P < 0.001, ****P < 0.0001. Supplementary Fig. 3. Fulvestrant can reverse the promoting effects of G3BP1 and G3BP1 Q392* on endometrial carcinoma. (A) HEC-1-A cells were transfected with indicator plasmids. The cells were cultured with DMSO and fulvestrant (100nM) in complete medium. 48h after transfection, cell lysates were prepared and the protein levels of ERα, SPOP and G3BP1 were determined by western blot. (B) CCK8 cell proliferation analysis was used to detect the proliferation ability of HEC-1-A cells. (C) Colony formation assay was used to detect t [file 12964_2023_1342_MOESM1_ESM.docx]
